# Multi-Etiological Hyponatremia in Association With Suspected Beer Potomania

**DOI:** 10.7759/cureus.36407

**Published:** 2023-03-20

**Authors:** Jayani Senanayake, Rangin Haji Rahman, Benoit Boucher, Muhammad Zain Ali, Sally Madanat, Carly Hammell, Francisco Chuy

**Affiliations:** 1 Medicine, Saint James School of Medicine, Anguilla, AIA; 2 Medicine, Saint James School of Medicine, St. Vincent, VCT; 3 Medicine, Washington University of Health and Science, Cleveland, USA; 4 Internal Medicine, Weiss Memorial Hospital, Chicago, USA

**Keywords:** congestive heart failure, acute euvolemic hyponatremia, beer potomania syndrome, osmotic demyelination syndrome (ods), clinician education, alcohol addictions, hypotonic hyponatremia, malnourishment, siadh, hyponatremia

## Abstract

Beer potomania is a unique condition characterized by hyponatremia secondary to excessive beer drinking and low daily solute intake. We report a case of a 41-year-old African American female with multiple comorbidities, notably alcohol use disorder, who was initially treated for hypertensive emergency and was subsequently found to be hyponatremic during the same visit. Beer potomania was suspected as a leading etiology of hyponatremia. This report emphasizes the importance of the proper diagnosis and appropriate management of beer potomania in the setting of concomitant comorbidities. Clinician awareness is crucial in implementing immediate treatment and in the prevention of potentially fatal sequelae such as severe malnutrition and osmotic demyelination syndrome.

## Introduction

Beer potomania is a syndrome of hyponatremia associated with excessive consumption of beer, in the absence of alternative causes of hyponatremia. First described in 1972, this condition is characterized by dilutional hyponatremia as a result of an exclusive beer diet that is devoid of salt and protein, as well as low daily solute intake [1,2]. Consequently, this reduces the solute load delivered to the kidneys, resulting in impaired water clearance and fluid retention, and subsequent hyponatremia [3]. At a physiologic level, there are two simultaneous processes occurring. The high carbohydrate content in beer prevents proteolysis from occurring, thus decreasing the generation of urea and thereby reducing the osmolar load to the kidneys [3]. That, coupled with the low solute content of alcohol and the poor dietary supplementation of sodium and potassium commonly seen in alcoholics, also contributes to reduced solute excretion and a state of fluid retention [3]. Furthermore, this condition increases the risk of rapid correction of hyponatremia, thereby increasing the likelihood of adverse neurological sequelae, namely, osmotic demyelination syndrome [4]. From a diagnostic perspective, beer potomania is difficult to recognize especially in the context of concomitant factors that impact body fluid status and sodium balance [3]. As such, a high index of clinical suspicion must be exercised to properly identify the underlying cause(s) of hyponatremia. This article presents a case of a 41-year-old alcoholic female who presented with euvolemic hyponatremia due to suspected beer potomania in the setting of multiple comorbidities.

## Case presentation

 A 41-year-old African American female patient was brought to the emergency department (ED) with altered mental status and alcohol intoxication. She has a past medical history significant for hypertension (HTN), hyperlipidemia (HLD), and a history of basal ganglia intracranial hemorrhage with right-side deficits. The patient also has a history of alcohol use disorder, consisting of her consuming large amounts of beer, and wine every day for 21 years. The patient’s family history includes diabetes and hypertension in her mother, heart disease in her father, and diabetes in her sister. On physical examination, the patient was not oriented to time and place and appeared thin and frail. There were decreased breath sounds present bilaterally in the lower lung lobes and a cardiac rub was appreciated on auscultation. The patient’s body mass index was 18.2 kg/m^2^ (N: 18.5-24.9kg/m^2^) and her blood pressure was found to be 190/112 mmHg (N: 120/80 mmHg). All other vital signs were unremarkable.

The patient was initially managed with lorazepam and supportive care for alcohol intoxication and prevention of withdrawal symptoms. Treatment was soon followed by nicardipine intravenous (IV) drips for her hypertensive emergency. Of note, the patient's outpatient medications included carvedilol and low-dose furosemide for hypertension, however, it is unclear whether she was compliant with these medications. Echocardiogram was then ordered for the patient, which demonstrated an ejection fraction of 20-25% with diffuse hypokinesis. Cardiology was soon consulted and guideline-directed medical therapy (GDMT) for heart failure management was initiated. Venous duplex ultrasound was performed to evaluate for deep vein thrombosis, which yielded negative results. The laboratory investigations performed at the ED are listed in Table 1.

**Table 1:** Laboratory workup performed at the initial visit to the emergency department GFR: glomerular filtration rate; BUN: blood urea nitrogen

Test	Result	Reference Range
Hemoglobin	9.3 g/dL	11-13.7 g/dL
Hematocrit	28.5%	34-44%
White blood cells	6.5x 10^2^/L	4.5- 11.0 x 10^2^/L
Platelets	165 x 10^9^/L	150-400 x 10^9^/L
Sodium	121 mEq/L	136- 146 mEq/L
Potassium	5.2 mEq/L	3.7-5.2 mEq/L
Chloride	88mEq/L	95-105 mEq/L
Serum alcohol	0.16%	< 0.05%
Glucose	97 mg/dL	70-100 mg/dL
Serum osmolality	265 mOsmol/kg H2O	275-295 mOsmol/kg H2O
Urine osmolality	309 mOsmol/kg H_2_O	50-1200 mOsmol/kg H_2_O
Urine sodium	60 mEq/day	40-220 mEq/day
GFR	60 ml/min/1.73 m^9^	>60 ml/min/1.73 m^9^
Albumin	3.1 g/dL	3.5-5.5 g/dL
BUN	19 mg/dL	6-24 mg/dL
Creatinine	1.02 mg/dL	0.35-1.35 mg/dL
High sensitivity troponin	9 pg/mL	≤ 15 pg/mL

Due to her hyponatremic state, nephrology was consulted and recommendations were made to increase oral (PO) intake of salt and protein and restrict water intake to 800 mL per day. Furthermore, basic metabolic panels were ordered every six hours to follow her sodium levels. Cardiology continued IV furosemide for one day and then planned to switch to oral therapy. Her sodium levels began trending back to normonatremia; however, she left against medical advice after three days. At discharge, the patient’s medications included folic acid, sacubitril-valsartan, thiamine, carvedilol, alprazolam, fluticasone propionate, pantoprazole, and sertraline. She was also recommended to discontinue furosemide and follow up with the cardiologist, nephrologist, and primary care physician. Notably, the patient is a poor historian and of low socioeconomic status.

Eighteen days following the patient’s leave against medical advice, she returned to the hospital with shortness of breath and chest pain that has been worsening over the last week. Her condition quickly deteriorated, and she was swiftly admitted to the floor where respiratory resuscitation was activated due to her becoming obtunded and sleepy. Her urine toxicology reports were positive for cannabis and amphetamines. She denied symptoms of fever or chills but admitted to consuming alcohol and tetrahydrocannabinol. The patient had not been following up with a primary care physician as of the previous hospital admission. On physical examination, she appeared mildly anxious, had mild conversational dyspnea, and distant lung crackles were appreciated at the lungs bases bilaterally. There was no stridor or use of accessory muscles. Her blood pressure was 131/94 mmHg, pulse 98 beats per minute (BPM) (N: 60-100 bpm), respiratory rate 20 breaths/min (N: 12-16 breaths/min), and oxygen saturation (SpO2) 98% (N: >95%). The patient appeared lethargic, but opened her eyes and followed commands when called multiple times. Pupils were 1 mm in size and reactive to light. A cardiac gallop was present without any friction rubs. The patient was quickly placed on a cardiac monitor and rhythm strip, which revealed a normal sinus rhythm. Electrocardiogram (EKG) also showed a sinus rhythm with narrow complexes, however, no acute ST changes or T wave abnormalities were evident. A chest X-ray (CXR), along with an ultrasound of the abdomen, was ordered. The patient’s CXR demonstrated signs of fluid overload, right basilar atelectasis/consolidation, and right pleural effusion. The patient was given a dose of IV furosemide for volume overload. The ultrasound of the abdomen showed hepatomegaly, moderate appearing ascites, and bilateral pleural effusions were noted incidentally. The sodium level obtained on the third day of admission was 118 mEq/L and the patient was transferred to the intensive care unit (ICU) for better management of the current condition. As per nephrology, the patient was treated with fluid restriction and kept on a low-sodium diet. Her repeated sodium levels gradually increased from 118 to 121 to 123 mEq/L the next day. On the fifth day of admission, the patient left against medical advice. The patient has not been readmitted as an inpatient as of the time of writing.

## Discussion

Hyponatremia is the most common electrolyte abnormality seen in hospitalized patients and is also a common presentation in patients with chronic alcoholism or acute alcohol intoxication [5]. Beer potomania is a unique syndrome of severe hyponatremia that results from extensive beer consumption and low solute intake. Alcohol, namely, beer, primarily consists of water and carbohydrates, with minimal amounts of electrolytes. The high carbohydrate content prevents muscular proteolysis, leading to a decrease in urea production and further contributes to a low solute content in the kidneys [2]. Subsequently, this results in a decreased clearance of free water that eventually results in dilutional hyponatremia. Patients typically present with altered mental status due to alcohol intoxication and low body weight from severe malnourishment, similar to the patient presented in this case. Many concomitant comorbidities, such as congestive heart failure, cirrhosis, and severe malnutrition, and the use of drugs such as thiazide diuretics, corticosteroids, and psychotropic drugs can exacerbate symptoms of beer potomania [2,6].

Currently, there are no specific diagnostic tests for beer potomania, thus it requires careful evaluation of a patient’s hyponatremic state. Hyponatremia is subdivided into isotonic hyponatremia, hypotonic hyponatremia, and hypertonic hyponatremia [7]. Serum osmolality of less than 275 mOsm/kg defines hypotonic hyponatremia, which is further divided into hypovolemic hyponatremia, euvolemic hyponatremia, and hypertonic hyponatremia based on the patient’s volume status [7]. Euvolemic hyponatremia occurs when there is an increase in total body water with normal total body sodium levels. Low urine osmolality raises suspicion for primary polydipsia and beer potomania [7]. In this patient, based on the low serum osmolality and low serum sodium levels, a diagnosis of hypotonic hyponatremia was made. Furthermore, based on clinical findings, such as normal skin turgor, absence of jugular venous distention, and peripheral edema, evaluated during the first admission, euvolemic hyponatremia was established. However, it is worth noting that our patient’s hypertensive emergency may have been due to her worsening congestive heart failure (CHF), possible medication nonadherence, and substance abuse. During history-taking, the patient denied a history of hypothyroidism, adrenal insufficiency as well as excessive thirst, and consuming large amounts of water, further ruling out primary polydipsia. Furthermore, she denied any history of dietary changes, ruling out tea and toast syndrome. Excessive fluid intake from consuming beer may result in a dilutional drop in hemoglobin and hematocrit levels, as was seen in our patient. Laboratory diagnostic criteria for beer potomania further include urine osmolality <100 mOsm/kg H2O and urine sodium <20 mmol/L [3]. This patient’s urine osmolality of 309 mOsmol/kg H2O and urine sodium of 60 mmol/L may also be suggestive of other concomitant comorbidities, causing an increase in her urine osmolality as well as her urinary sodium excretion. A case study by Bhattarai et al. explains that urine osmolality is an inconsistent finding in patients with beer potomania and can even be abnormally high in some cases due to alcohol-induced antidiuretic hormone (ADH) suppression as well as excess alcohol in urine masking the hypotonicity of urine [8]. It is also worth mentioning that our patient’s high urine sodium excretion (>40 mmol/L) may have been a result of furosemide-induced diuresis [3]. However, given this patient’s malnourishment, chronic history of alcohol use, and recent alcohol intoxication, she was at an increased risk of developing beer potomania. Further investigations are crucial for a proper diagnosis and treatment of all underlying conditions and correction of hyponatremia.

Treatment of beer potomania must be initiated immediately when suspicions arise. Initial management includes fluid restriction, obtaining serial serum sodium levels, and the administration of intravenous (IV) dextrose if the patient is calorie deficient. The target goal for serum sodium levels includes increasing serum sodium levels to <10 mEq/L in the first 24 hours and <18 mEq/L in the first 48 hours [9]. Careful management is necessary, as rapid correction of hyponatremia can result in osmotic demyelination syndrome. Because of these potential complications, it is important to treat patients in the intensive care unit for better management of beer potomania [9]. Notably, potassium levels should also be closely monitored to avoid hypokalemia, especially in patients treated with diuretics such as our patient [6]. Uncorrected hyponatremia may lead to serious complications, such as seizures, coma, rhabdomyolysis, and altered mental status, and further encourages treatment compliance in such patients [7]. Lastly, patients must be advised on the importance of alcohol cessation and appropriate nutrition intake.

Syndrome of inappropriate antidiuretic hormone secretion (SIADH) is a differential diagnosis that exhibits a similar clinical presentation to beer potomania. This condition can be seen in central nervous system (CNS) disturbances, malignancies, ADH hormone deficiency and administration, and in the setting of human immunodeficiency virus infection [10]. Pathologically, SIADH involves excessive ADH-induced water retention leading to loss of solutes in the kidneys. This creates a euvolemic, hypotonic hyponatremic state [10]. Diagnosis of SIADH follows a similar pattern to that of beer potomania and, in addition, requires the exclusion of other possible causes of hyponatremia. Treatment of SIADH varies according to the severity of the disease. For mild symptoms, fluid restriction may be sufficient while severe disease may require ADH antagonists such as conivaptan or tolvaptan. Our patient’s chest CXR did not demonstrate masses concerning for malignancy (thus ruling out SIADH due to a paraneoplastic syndrome); however, her CXR was significant for multiple cardiac and pulmonary disease processes, possibly raising suspicion for a concomitant SIADH. In addition, her history of stroke and use of selective serotonin reuptake inhibitors (SSRIs) may suggest a diagnosis of possible SIADH [10]. Notably, the improvement in serum sodium level after treatment with fluid restriction may be suggestive of mild symptoms of SIADH coexisting with beer potomania. Moreover, SSRI use has also been documented to be associated with hyponatremia, especially at the beginning of treatment initiation, possibly through the inhibition of norepinephrine reuptake [11]. However, this patient's SSRI treatment initiation is not known due to limitations to history intake. Regardless, it is of the utmost importance to recognize the accompanying causes of hyponatremia to guide the proper management and care of patients.

Furthermore, our patient’s low ejection fraction of 20-25%, in conjunction with ventricular hypokinesis, shortness of breath, and pulmonary changes, is suggestive of CHF. Additionally, abdominal ultrasound showing hepatomegaly and a moderate amount of ascites is suggestive of liver disease in this patient. Cirrhosis and CHF can lead to hypervolemic, hypotonic hyponatremia [7]. The patient’s clinical presentation during the second admission can therefore be attributed to the disease processes of cirrhosis and CHF, however, concomitant diagnoses warrant diagnostic workup in order to mitigate the risk of future complications. Despite the presence of multiple disease processes, it is reasonable to ascribe one of the leading differentials of her hyponatremia to beer potomania on account of her long history and accompanying clinical presentation. Nevertheless, clinician awareness, proper diagnosis, and adequate management along with verification of treatment compliance of beer potomania are essential steps in the prevention of consequential complications.

## Conclusions

In conclusion, this study outlined a case of a 41-year-old alcoholic female who presented with euvolemic hyponatremia due to suspected beer potomania in the setting of multiple comorbidities. The patient’s history of alcohol consumption, euvolemic clinical state on admission, and rise in sodium levels in response to water restriction to 800 mL per day led clinicians to suspect that beer potomania was a leading differential diagnosis. With a review of the literature, we discussed the importance of judiciously correcting hyponatremia in beer potomania to prevent complications, such as osmotic demyelination syndrome, as an underlying state predisposes them to a higher risk of serious neurological sequelae. Finally, the case emphasizes the importance of clinician understanding of the pathophysiology of beer potomania in order to tailor management in the context of accompanying comorbidities and to further mitigate disease progression.
